# Endoscopic release of congenital muscular torticollis with radiofrequency in teenagers

**DOI:** 10.1186/s13018-018-0801-6

**Published:** 2018-05-03

**Authors:** Jun-liang Wang, Wei Qi, Yu-jie Liu

**Affiliations:** 1 Department of Orthopedics, Hainan Branch of Chinese PLA General Hospital, Haitang District, Sanya, 572000 Hainan Province China; 2 Department of Orthopedics, Chinese PLA General Hospital, Beijing, 100853 China

**Keywords:** Congenital muscular torticollis, Arthroscopy, Local anesthesia, Radiofrequency

## Abstract

**Background:**

Congenital muscular torticollis (CMT) is due to contracture of the sternocleidomastoid muscle which may cause activity limitations of the neck, tilt of the head, craniofacial asymmetry, and deformity of the skull. The authors present their experience of arthroscopic tight fibrous band release with radiofrequency in teenagers under local anesthesia and evaluate the clinical results.

**Methods:**

A total of 69 patients who underwent arthroscopic release of CMT with radiofrequency under local anesthesia by a single surgeon could participate in this study. Before operation, surface landmarks of sternocleidomastoid muscle, bone, and neurovascular structures were marked. Local infiltrating anesthesia of the surgical region was then performed. Through a working space created by blunt dissection, the arthroscopy and radiofrequency devices were introduced. Then, the clavicular and sternal heads of the sternocleidomastoid muscle were identified and gradually transected. The patients were followed up postoperatively with Cheng’s scoring system.

**Results:**

There were 31 male patients and 38 female patients. The mean age of the patients was 16.1 years. The mean length of follow-up in this series was 36.7 months (range, 28 to 67 months). During the operation, 62 patients (89.9%) had no pain, 6 patients (8.7%) felt mild pain, and only 1 patient (1.4%) regarded the procedure as very painful. At all follow-up periods, there were no repeat arthroscopies for any of these patients. At the final follow-up, the average rotation deficit improved from 22.5° to 4.1° postoperatively, and the average lateral bending deficit improved from 14.6° to 3.3° (*p* < 0.05). Overall, the clinical result was good or excellent in 65 patients (94.2%), fair in 4 patients, and poor in 0 patients within the follow-up period according to Cheng’s scoring system. To date, no patients had any intraoperative or postoperative complications from this procedure.

**Conclusion:**

The arthroscopic release with radiofrequency under local anesthesia provides surgeons with an alternative to traditional open techniques for the management of congenital muscular torticollis (CMT). Our date shows that this method is minimally invasive and provides good functional recovery with a lower risk of complications.

## Background

Congenital muscular torticollis (CMT) is a relatively common condition caused by contracture of the sternocleidomastoid muscle, which may cause activity limitations of the neck, tilt of the head, craniofacial asymmetry, and deformity of the skull [1, 2]. Applications of an orthosis, program of stimulation exercise and positioning, and manual stretching have been recommended as non-operative treatment [3]. If those treatments fail, surgical release of the tight fibrous band is frequently required.

As the surgical techniques are constantly being advanced and modified, different operative methods and approaches have been advocated. Burstein described the original technique of endoscopic release for CMT for the first time in 1998 [4]. Several authors then reported different endoscopic release techniques for CMT [5–10]. The well-known advantage of endoscopy in contrast to the open operation is less traumatized and quick rehabilitation. In previous studies, endoscopic surgeries were done under general anesthesia. Nonetheless, to our knowledge, arthroscopic release of CMT with radiofrequency under local anesthesia has not been reported in a series of patients.

The purpose of the study was to present a reliable arthroscopic technique for the treatment of CMT with radiofrequency under local anesthesia and report the clinical results at a minimum 2-year follow-up in our institution.

## Methods

### Patients

Between March 2003 and May 2014, 69 patients who underwent arthroscopic treatment of CMT under local anesthesia by the senior author (L.Y.J) were able to participate in this study. The inclusion criteria consisted of patients with congenital muscular torticollis, who were 12–19 years old, and deficits of rotation and lateral bending of the neck > 15°. All the patients had failed in different forms of conservative treatment consisting of application of orthosis, manual stretching, and physical therapy. No patients had undergone surgery previously. Spasmodic torticollis caused by disorder of the central nervous system, torticollis caused by deformity of the cervical spine, and severe CMT which has caused the deformity of the cervical spine were excluded.

All patients were asked to complete a preoperative questionnaire that includes their demographic and detailed medical history. The degree of head tilt, the range of rotation and lateral bending of the head, and facial asymmetries were recorded. Posteroanterior view radiographs were obtained preoperatively to exclude the severe deformity of the cervical spine. Patients were provided informed consent for the operation and followed our rehabilitation protocol. This observational, therapeutic case study was approved by the institutional review board of our hospital.

### Surgical procedure and postoperative care

**Fig. 1:**
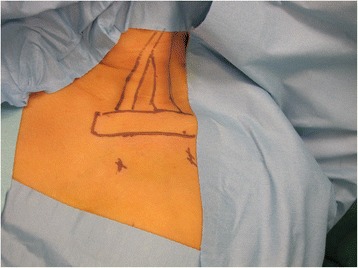
The SCM muscle was placed under tension by extension of the neck and rotation of the head toward the unaffected side. The anteromedial portal is placed at 3 cm inferior and 1.5 cm medial to the sternoclavicular joint of the affected side, and the working portal is placed at 3 cm inferior to the midpoint of the clavicle

All patients are positioned in a supine position with extension of the neck and rotation of the head towards the unaffected side. Surface landmarks of sternocleidomastoid muscle, clavicle, neurovascular structures, and arthroscopic portals are identified by careful palpation and marked using a surgical marker. Two portals are typically used, including the anterolateral working portals and the anteromedial portal which accommodates the arthroscopy. The anteromedial portal is placed at 3 cm inferior and 1.5 cm medial to the sternoclavicular joint of the affected side, and the working portal is placed at 3 cm inferior to the midpoint of the clavicle (Fig. 1). Routine sterile preparation and drape are then performed. Local infiltrating anesthesia of the portal sites and the surgical region is performed with 10 ml 2% lidocaine, which is diluted with 30 ml saline water.

**Fig. 2:**
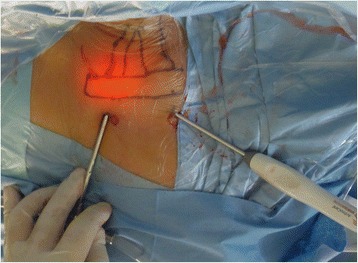
Picture showing the arthroscope and the radiofrequency probe insertion for operation

**Fig. 3:**
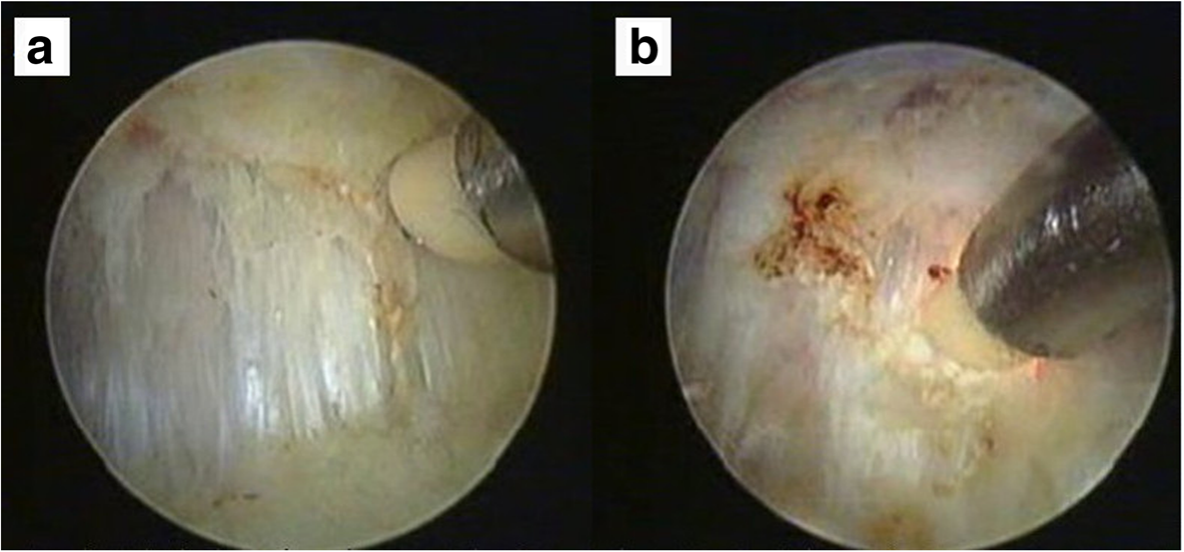
The tight band was identified (**a**) and gradually transected from superior to inferior with the radiofrequency probe (**b**)

After adequate anesthesia was obtained, a 2- to 3-mm transverse incision was made just over the marking portals with a No. 11 scalpel. The subcutaneous tissue was blunt-dissected from underlying structures with a periosteal elevator to create a working space. The mean size of the working space was 5 × 6 cm. Normal saline (containing 1 mg adrenaline per 3000 mL normal saline) subsequently was injected. This created a relatively bloodless working space. After a 30° 4-mm arthroscopy and a radiofrequency probe (ArthroCare Atlas System with TriStar 50 ArthroWand; ArthroCare Corporation, Sunnyvale, CA) were introduced to the working space, we clean up the fibrous tissue affecting the range of vision (Fig. 2). Patients were asked to hyperextend and rotate the head to tense muscles, and then, the clavicular and sternal heads of the sternocleidomastoid muscle were identified. With the radiofrequency probe, we gradually transected from superior to inferior in the insertion regions (Fig. 3). During arthroscopic-assisted release, care was taken not to resect too deep and thus stay clear of the neurovascular structures. Any bleeding point was coagulated using radiofrequency energy to maintain a clear vision and prevent a hematoma formation after the operation. Before completing the arthroscopic release, patients were requested to laterally bend and rotate the neck towards the contralateral side to evaluate the degree of release and not to leave a residue of contracture. During surgery, blood pressure, heart rate, and blood oxygen saturation of the patients were regularly monitored.

Immediately postoperatively, all patients participated in a uniform rehabilitation protocol. No brace was needed. Gentle ROM exercises and strengthening exercises were initiated from the first postoperative day and continued for 4 to 6 weeks after surgery.

### Outcome assessment

Two independent observers (W.J.L, Q.W) performed the clinical examination in all patients preoperatively and then each time of follow-up. Clinical assessments were based on Cheng’s scoring system [11], which includes both subjective and objective criteria. The evaluation category includes rotation deficit, lateral bending deficit, craniofacial asymmetry, residual band, head tilt, and subjective assessment by parents. Each category is scaled into four levels according to the severity and marked as 0, 1, 2, and 3 points. Cheng’s scoring system is scaled between 1 and 18, where lower scores represent more disability. According to Cheng’s scoring system, an overall score of 16, 17, or 18 points indicates an excellent result; 12 to 15 points, a good result; 6 to 11 points, a fair result; and < 6 points, a poor result.

### Statistical analysis

Statistical analysis was performed with SPSS software (version 17.0; SPSS, Chicago, IL). All quantitative data are expressed as means ± standard deviation (SD). Statistical analysis was performed using a rank sum test to compare preoperative and postoperative results for rotation deficit and lateral bending deficit. The level of significance was defined as *p* = 0.05.

## Results

By the time of data collection for this report, all of 69 patients operated on were followed up. The mean follow-up period was 36.7 months (range, 28 to 67 months). There were 31 male patients (44.9%) and 38 female patients (55.1%) with an average age of 16.1 ± 1.6 years (range, 13–19 years). The CMT occurred in the left side in 47.8% (*n* = 33) and in the right side in 52.2% (*n* = 36). There was no patient who manifested bilateral torticollis. Table 1 shows the study population data.

**Table 1:** Descriptive statistics of the study population

	Data
No. of patients	69
No. of patients available for follow-up	69
Follow-up interval (months)	36.7 ± 8.4 (28–67)
Age at surgery (years)	16.1 ± 1.6 (13–19)
Gender (M/F)	31/38
Side(R/L)	36/33

Data are given as mean ± SD (range) unless otherwise indicated

The operation was successfully completed in all patients. No repeat arthroscopy was needed for all patients. During the operation, 62 patients (89.9%) had no pain, 6 patients (8.7%) felt mild pain, and only 1 patient (1.4%) regarded the procedure as very painful.

**Fig. 4:**
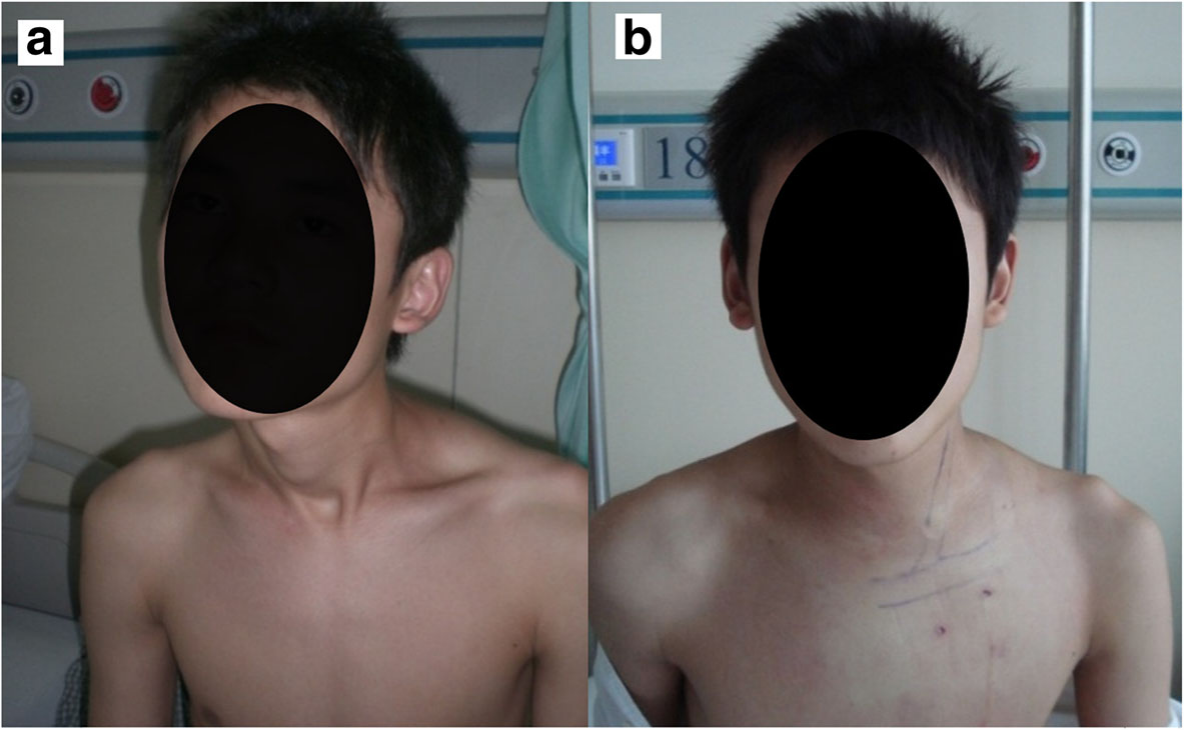
Preoperative view (**a**) of a 15-year-old boy with left-sided torticollis. Appearance at 2 days after surgery (**b**)

There was no residual band and head tilt (Fig. 4). At the final follow-up, the average rotation deficit improved from 22.5° to 4.1° postoperatively, and the average lateral bending deficit improved from 14.6° to 3.3° (*p* < 0.05). The clinical result was good or excellent in 65 patients (94.2%), fair in 4 patients, and poor in 0 patients within the follow-up period according to Cheng’s scoring system [11]. None of the patients had neurovascular injuries, hematomas, or wound infections during the follow-up period, and no remaining fibrotic band or tightness was detected.

## Discussion

The present study described a new arthroscopic technique for the treatment of CMT under local anesthesia with a radiofrequency probe. In this study, we found the arthroscopic technique provided satisfactory and clear surgical vision. Local anesthesia provided adequate analgesia which ensures smooth operation. The clinical results have shown that arthroscopic release of CMT under local anesthesia provided a satisfactory function and excellent cosmetology effect.

Different conservative and surgical treatments have been reported for CMT. However, conservative treatments are more suitable for infants and small children [12–15]. We do believe that observation and physical therapy is usually an effective treatment in most cases, especially if instituted within the first year of life [16]. In literature, good and excellent clinical results after manual stretching have been reported in the young patient [17]. Luther reported stretching exercises was the most common form of treatment with positive outcomes for over 90% of the identified cases [18]. Nevertheless, if the conservative treatments fail, surgery should be discussed. Surgical procedures include unipolar release, bipolar release, and endoscopic release. However, the issue of the most appropriate surgical approach remains controversial.

At present, the appropriate age for the surgery is still a matter of debate. Shim recommended that operative treatment for congenital muscular torticollis should be postponed until the patient can comply successfully with post-operative bracing and an exercise program [19]. However, other authors believed that surgery should be carried out as soon as possible. Wirth recommended that a biterminal release be performed at the age of 3 to 5 years in all patients who do not respond to non-operative treatment [20]. Akazawa reported partial resections of the sternocleidomastoid muscle in 38 patients of CMT. Good results were obtained in 90% of patients under 5 years old and in 50% of patients aged 6 years or more at the operation [21]. Sonmez considered those patients whose pathology does not resolve after 12 months of physical therapy or who develop facial asymmetry or plagiocephaly during the follow-up period should be operated on in order to achieve the best cosmetic result [22].

Traditionally, contracture release of congenital muscular torticollis has been done in an open manner. And reduction of the contractures and improvement of function with open surgery had been reported [23–25]. Patwardhan reported a bipolar release of sternocleidomastoid and Z-lengthening in 12 adult patients, and eight patients had excellent results and four had good results according to Cheng’s scoring system [24]. Lee reported a series of 20 patients underwent complete tight fibrous band release and resection. Eighteen patients had a full range of motion of neck rotation and lateral flexion; only one patient showed a 10° limitation in lateral flexion, and one showed 10° limitations of neck rotation and lateral flexion [25].

However, traditional open operation is more invasive and causes a permanent scar in the neck, which does not meet the high esthetic demands of patients with CMT.

In 1998, Burstein originally described the technique of endoscopic release for CMT using a retroauricular endoscopic access point [4]. The well-known advantage of endoscopy in contrast to open manner is that the soft tissue is minimally invasive. Nevertheless, in our opinion, this technology has a higher risk of nerve injury. Dutta and Tang et al. reported their technique of transaxillary subcutaneous endoscopic release of the sternocleidomastoid muscle for treatment of CMT [7, 9, 10, 26]. Because the incision was made in the anterior axillary fold, a long subcutaneous tunnel over the clavicular and sternal heads of the SCM muscle was needed. Therefore, the transaxillary approach is usually carried out under general anesthesia. Therefore, the surgeon needed to decide the precise scope and extent of release based on experience.

Our surgical method is characterized by arthroscopic repair under local anesthesia with radiofrequency energy. The approach in our method is directly designed around the operating area using micro-neck incision and provides a more minimal invasion compared with the transaxillary approach. Under local anesthesia, the patients can rotate or bend the head with a surgeon’s instruction; therefore, real-time release effect could be evaluated. However, for the infants and young children who cannot cooperate with surgery, we still recommend to take the operation under general anesthesia. Because conventional electrosurgical devices remove target tissues by rapid heating or burning, the surrounding normal tissue may be damaged. However, RF devices gasified the target tissue at a relatively low temperature; the surgery was safer.

This study evaluated the feasibility and efficacy of arthroscopic release of CMT with radiofrequency under local anesthesia in a cohort of patients. The intermediate results of this technique used for CMT are encouraging. The patient satisfaction rate was relatively high. The mean Cheng’s scoring system for the surgically treated patients were statistically improved at mean 36.7 months follow-up. At follow-up, 94.2% of the patients rated as either good or excellent. In this study and in our clinical experience, no severe complications had occurred, so we believe our surgical methods are clinically feasible and efficient.

There were several limitations to our study. The most important limitation was that it was a retrospective design. Second, we do not have a control group of patients treated by other surgical methods. Therefore, we cannot make sure that this surgical method provided fewer complications and better outcomes than an open manner. Despite the limitations of this study design, our series was fairly large with 69 patients and the follow-up period was relatively long of 36.7 months. Thus, results of this study add valuable information in the treatment of CMT. In the future, randomized controlled trials to compare conservative treatment and different surgical methods will be needed to determine which treatment is related to the better results, the lower morbidity, and higher patient satisfaction.

## Conclusions

Our study supports the hypothesis that arthroscopic release under local anesthesia is a safe and reliable treatment for CMT in selected cases of congenital muscular torticollis. This technique may provide patients with improved function and excellent cosmetology effect.

## Abbreviations

CMT: Congenital muscular torticollis
RF: Radiofrequency
ROM: Range of motion
SD: Standard deviation
